# Pain-related sensory innervation in monoiodoacetate-induced osteoarthritis in rat knees that gradually develops neuronal injury in addition to inflammatory pain

**DOI:** 10.1186/1471-2474-12-134

**Published:** 2011-06-16

**Authors:** Sumihisa Orita, Tetsuhiro Ishikawa, Masayuki Miyagi, Nobuyasu Ochiai, Gen Inoue, Yawara Eguchi, Hiroto Kamoda, Gen Arai, Tomoaki Toyone, Yasuchika Aoki, Takekazu Kubo, Kazuhisa Takahashi, Seiji Ohtori

**Affiliations:** 1 Department of Orthopaedic Surgery, Graduate School of Medicine, Chiba University, Chiba, Japan; 2 Department of Orthopaedic Surgery, Chiba Rosai Hospital, Chiba, Japan; 3 Teikyo University Chiba Medical Center, Chiba, Japan; 4 Department of Neurobiology, Graduate School of Medicine, Chiba University, Chiba, Japan

## Abstract

**Background:**

The exact mechanism of knee osteoarthritis (OA)-associated pain is unclear, whereas mixed evidence of inflammatory pain and neuropathic pain has been noted. We aimed to investigate pain-related sensory innervation in a monoiodoacetate (MIA)-induced model of OA.

**Methods:**

Sixty of seventy female Sprague Dawley rats of six week-old underwent intra-articular MIA and fluorogold (FG) retrograde neurotracer injection into their right (ipsilateral) knee, while their left knees were treated with FG in saline as a control (contralateral knee). Other rats were treated with FG only bilaterally, and used as controls. Rats were evaluated for tactile allodynia using von Frey hairs. Proinflammatory mediators in the knee soft tissues, including tumor necrosis factor (TNF)-α, interleukin (IL)-6, and nerve growth factor (NGF), were quantified using ELISAs to evaluate inflammation in the knee after 1, 4, 7,14,21, and 28 days post injection:. Dorsal root ganglia (DRG) were immunostained for three molecules after 7,14,21, and 28 days post injection: calcitonin gene-related peptide (CGRP), a marker of inflammatory pain; and activating transcription factor-3 (ATF3) and growth associated protein-43 (GAP43), as markers for nerve injury and regenerating axons. The distribution of microglia in the spinal cord were also evaluated, because they have been reported to increase in neuropathic pain states. These evaluations were performed up to 28 days postinjection. *P *< 0.05 was considered significant.

**Results:**

Progressive tactile allodynia and elevated cytokine concentrations were observed in ipsilateral knees. CGRP-immunoreactive (-ir) ipsilateral DRG neurons significantly increased, peaking at 14 days postinjection, while expression of FG-labeled ATF3-ir or ATF3-ir GAP43-ir DRG neurons significantly increased in a time-dependent manner. Significant proliferation of microglia were found with time in the ipsilateral dorsal horn.

**Conclusions:**

Pain-related characteristics in a MIA-induced rat OA model can originate from an inflammatory pain state induced by the local inflammation initiated by inflammatory cytokines, and that state will be followed by gradual initiation of neuronal injury, which may induce the neuropathic pain state.

## Background

Knee osteoarthritis (OA) is a common chronic degenerative disease characterized by loss of articular cartilage components [1], affecting the entire joint structure including the synovial membrane, fat pad, and subchondral bone. The main clinical sign of OA is joint pain, which has a yet to be clarified mechanism, whereas several animal models have been developed for OA research. The pathological models for OA include intraarticular injection of monoiodoacetate (MIA) into the rat femorotibial joint space, which has been reported to produce a linear pathology with similarities to OA [2,3] and significant pain-related behavior [4,5]. Regarding the characteristics of the pain, a previous study has indicated the coexistence of an inflammatory pain state, chemically initiated by inflammatory mediators, and a neuropathic pain state, physically initiated by nerve injury, in the models [6]. However, the details of these pain states are unclear. One of the sources of the pain is considered to be local inflammation in knee joints, such as that in the synovial membrane that is considered to promote pathological OA [7-9] followed by the production of several cytokines such as tumor necrosis factor (TNF)-α, interleukins (IL) such as IL-1β and IL-6, and nerve growth factor (NGF). These mediators further contribute to OA pathogenesis by increasing cartilage degradation and inducing hyperalgesia. TNF-α activates sensory neurons directly via its receptors and initiates a cascade of inflammatory reactions through the production of ILs such as IL-1 and IL-6 [10,11]. In particular, IL-6 is reported to have a complex role in OA pathogenesis by initiating inflammatory responses such as the production of tissue inhibitors of metalloproteinases-1, which may act to limit cartilage damage through negative feedback [12]. NGF is reported to be upregulated in human osteoarthritic chondrocytes and synovial fibroblasts, suggesting its important role in OA pathology [13,14].

To describe inflammatory-related pain, evaluation at the level of dorsal root ganglia (DRG) is also important. DRG neurons are divided into three classes: large DRG neurons responsible for proprioception [15], small DRG neurons containing pain-related neuropeptides such as substance P and calcitonin gene-related peptide (CGRP) [16-18], and small DRG neurons without these neuropeptides that can be labeled with isolectin-B4 (IB4), a structural protein from *Griffonia simplicifolia *[17,18]. CGRP has been reported to produce hyperalgesia, and its elevation is suggested to produce a pain sensation derived from inflammation [19]. Its expression in DRG neurons has been reported to be increased in MIA-induced OA models [20,21], although its time course has yet to be reported. Other studies imply the existence of nerve injury that may lead to neuropathic pain, showing the increased expression of activating transcription factor (ATF)-3-immunoreactive (ir) DRG neurons by using rat MIA-induced OA models [22]. ATF3 is reported to be a selective marker of cell damage following nerve injury [23]. To clarify their association with nerve injury, evaluating the expression of growth-associated protein (GAP)-43 and ATF3 at the level of the DRG may be useful because these molecules are elevated in DRG neurons with regenerating axon fibers after nerve injury, which may indicate the pathogenesis of neuropathic pain. However, the expression of these molecules has also not been investigated. Furthermore, it may be important to examine at the level of the spinal cord where glial cells such as microglia are reported to play an important role in facilitating neuropathic pain derived from neural injury followed by their proliferation, morphological changes, and increased expression of glial markers, such as the ionized-calcium-binding adapter molecule (Iba)-1 [24-26]. It has also been reported that CGRP-containing sensory nerve fibers emerge in the deepest layer of the dorsal horn of the spinal cord [27]. However, as far as we know, no related studies have evaluated the time course of these findings in spinal cords of animals in OA models.

The present study aimed to investigate the time course of pain-related mediators, particularly focusing on both inflammatory and neuropathic pain-related states by using local tissues and sensory innervation of the peripheral nervous system including DRG and spinal cords of an MIA-induced model of OA in rats.

## Methods

All protocols for animal procedures were reviewed and approved by the ethics committee of our institution and followed the National Institutes of Health Guidelines for the Care and Use of Laboratory Animals (1996 revision).

### Intraarticular injection of MIA and retrograde neurotracer

Seventy female Sprague-Dawley rats weighing 200-300 g at 6 weeks of age were prepared (CLEA, Tokyo, Japan). All rats were anesthetized with an intraperitoneal (i.p.) injection of sodium pentobarbital (40 mg/kg) and treated aseptically. For 60 animals their right knees were treated with a single intraarticular injection of 2 mg of MIA (Sigma-Aldrich, St. Louis, MO) and 2% of the retrograde neurotracer FG (Fluorochrome, Denver, CO) in 25 μl of sterile saline. The solution was injected through the patellar ligament by using a 27G needle with the leg flexed at a 90° angle at the knee. The left knees were treated with FG alone. The doses of the drugs were based on previous literature [28,29]. Subsequently, right knees with an intraarticular injection of MIA were identified as the "ipsilateral" side, whereas the left knees were "contralateral." The contralateral knee joints were used as controls in the following experiments. The 10 animals left were treated with FG bilaterally, and used as controls.

### Behavioral testing for tactile allodynia (von Frey hairs)

Tactile allodynia was assessed by measuring withdrawal thresholds to calibrated von Frey hairs just before the injection and at points 1, 4, 7, 14, 21, and 28 days after the intraarticular injection as done previously [6,28]. The test was performed by touching the plantar surface of the hind paws of awake animals placed into a chamber with a metal grid floor with von Frey hairs in ascending order of force. A positive response was noted if the paw was sharply withdrawn or there was flinching upon application of the hair. Once a positive withdrawal response was established, the paw was retested, starting with the next descending von Frey hair until no response occurred. The lowest amount of force required to elicit a response was recorded as the PWT (in grams). Statistical analysis for behavioral experiments was conducted on raw data by using repeated measures analysis of variance (ANOVA) followed by Tukey's post-hoc test (*P *< 0.05 was identified as significant).

### Tissue preparation

After deep anesthesia, the soft tissues around each side of the joint and cartilage including the synovium and capsule were resected (n = 7, each). The other rats were left with their bilateral knees untreated for histopathologic examination by using hematoxylin and eosin (H-E) staining. The rats were then perfused transcardially with 0.9% saline, followed by 500 ml of 4% paraformaldehyde in phosphate buffer fixative (0.1 M, pH 7.4). Bilateral untreated limbs, bilateral L4 DRGs, and spinal cord at the level of the lumbar enlargement were resected. The resected limbs were prepared for histopathology, and the DRG and spinal cord specimens were prepared for immunohistology.

### Histopathology of the knee joint

The resected limbs were cut at midfemur and midtibia and immersed in buffered paraformaldehyde fixative at 4°C for 1 week. The specimens were continuously demineralized in 10% EDTA for 2 weeks followed by standard histological techniques by using paraffin blocks for subsequent coronal (dorsoventral) sectioning. The samples were serially sectioned in steps of 8 μm, stained using H-E, and assessed by light microscopy.

### ELISA of the local tissues

Samples obtained just before the injection (control) and at points 1, 4, 7, 14, 21, and 28 days after the intraarticular injection and were collected (n = 7, each). The trimmed samples of soft tissue inside the knee, proliferated synovial membrane and anterior to the lateral capsule of the ipsilateral knees and anterior to the lateral capsule of the contralateral knees, were cut into pieces about 1 mm × 1 mm × 2 mm in size, well-rinsed in PBS, frozen in liquid nitrogen, and then crushed into powder and homogenized in a 200-μl lysis/extraction reagent (Sigma-Aldrich). The samples were centrifuged at 14,000 rpm for 10 min at 4°C, and then the supernatants were extracted for the assay. The concentrations of the cytokines were measured using ELISA kits optimized for each cytokine following each manufacturer's protocol as follows: IL-6 (R&D Systems, Minneapolis, MN), TNF-α (R&D Systems), and NGF (Millipore, Temecula, CA). The limits of sensitivity for the cytokines measured were 14, < 5, and 10 pg/ml, respectively. The total protein concentration in all samples was measured using the Lowry method by using a DC protein assay kit (Bio-Rad Laboratories, Hercules, CA).

After the measurement with a microplate reader (Iwaki, Tokyo, Japan), we converted the absolute cytokine concentrations into corresponding concentrations per mg total protein. We statistically evaluated the cytokine concentrations by using nonrepeated ANOVA followed by an SNK post hoc test.

### IHC of the DRG and spinal cord specimens

Samples were prepared 7, 14, 21, and 28 days after the intraarticular injection including control. The DRG and spinal cord specimens were immersed in a buffered paraformaldehyde fixative at 4°C overnight and then immersed in PBS containing 20% sucrose for 20 h at 4°C. After freezing in liquid nitrogen, each specimen was sectioned at 10-μm thickness on a cryostat (Leica Microsystems, CM3050S, Wetzlar, Germany). The specimens were then treated for 90 min at room temperature with a blocking solution consisting of PBS containing 0.3% Triton X-100 and 3% skim milk.

The DRG specimens were processed using a rabbit antibody against CGRP (1:1000; Immunostar, Hudson, WI) and a biotin-labeled antibody against IB4 (1:1000; Molecular Probes, Eugene, OR) or a rabbit antibody against ATF3 (1:50; Santa Cruz, Delaware, CA) and a mouse antibody against GAP43 (1:1000, Millipore). The spinal cord specimens were processed using a rabbit antibody against Iba-1 (1:1000; Wako, Osaka, Japan).

After incubation with the diluted antibodies for 20 h at 4°C, DRG sections were incubated with Alexa 488-conjugated goat anti-rabbit IgG (for CGRP immunoreactivity, 1:1000; Molecular Probes) and Alexa 594-streptavidin conjugates (for IB4 binding, 1:1000; Molecular Probes) or Alexa 488-conjugated goat anti-rabbit IgG (for ATF3 immunoreactivity, 1:1000) combined with Alexa 594-conjugated goat anti-mouse IgG (forGAP43 immunoreactivity, 1:1000; Molecular Probes). Spinal cord specimens were incubated with Alexa 488-conjugated goat anti-rabbit IgG (for Iba-1 immunoreactivity, 1:1000). After each step, the sections were rinsed three times in PBS. The immunostained sections were observed using a fluorescence microscope (Olympus, Tokyo, Japan) in a treatment-blinded manner. The numbers of FG-labeled CGRP-ir or IB4-binding DRG neurons and FG-labeled ATF3-ir or ATF3-ir GAP43-ir DRG neurons in the DRG sections were counted. Their proportion to the total number of FG-labeled DRG neurons was calculated respectively for each DRG sample. The density of Iba-1-ir microglia per 100 mm^2 ^was calculated for both ipsilateral and contralateral dorsal horns. We statistically evaluated the distribution of immunostained DRG neurons by using non-repeated ANOVA with a post hoc SNK test and evaluated the distribution of microglia by using a Mann-Whitney *U *test.

## Results

### Confirmation of rat knee OA models induced by MIA injection

**Figure 1:**
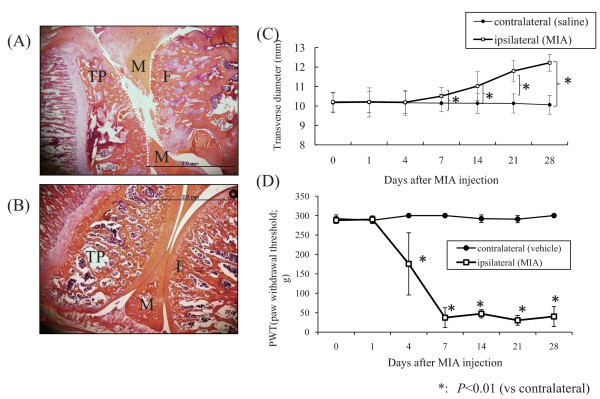
**Histological and behavioral evaluation**. Histological H-E stained appearance of the epiphysis 28 days after intraarticular injection: (A) ipsilateral and (B) contralateral. The scale bars are 2.0 mm. F: femoral condyle; T: tibial condyle, M: meniscus. The histological examination revealed OA-like findings, such as an extensive area of cartilage loss and degeneration, subchondral bone collapse, and moderately well-defined subchondral cystic structure, whereas that of the contralateral knee yielded normal findings. (C) Transverse diameter of the knees. The measured diameter showed significant gradual increase in the ipsilateral knees as the postinjection period passed (*P *< 0.05). (D) Behavioral profile in the von Frey hairs test. PWT; paw withdrawal threshold. Each point represents the mean value ± S.E. PWT significantly decreased after MIA injection in the ipsilateral limbs from post-injection day 4 and showed a similar tendency to decrease from postinjection day 7 to 28.

The average weight of the animals was 275.3 ± 12.2 g without any significant bias. All of the ipsilateral knees treated with MIA showed OA-like changes with macroscopic swelling, deformity. The contralateral vehicle-treated knees showed no changes following the injection of FG in sterile saline. The histological appearance of MIA-injected knees showed OA-like appearances such as an extensive area of cartilage loss and degeneration, subchondral bone collapse, and moderately well-defined subchondral cystic structure (Figure 1A), whereas the contralateral knees showed a normal appearance (Figure 1B). Furthermore, the measured diameters of the knees at each time point showed significant increases after day 7 (Figure 1(C)) (*P *< 0.05)

### Behavioral testing for tactile allodynia (von Frey hairs)

We examined the behavioral profile for von Frey hair testing, which records the lowest amount of force required to elicit a response to needle stimulation on the paw of the animals (paw withdrawal threshold, PWT; in grams). Figure 1D shows the behavioral profiles of the ipsilateral and contralateral limbs. PWT was significantly decreased beginning on postinjection day 4 in the MIA-treated ipsilateral limbs (*P *< 0.01), although PWT did not significantly changed from day 7 through the end of the experimental period. The vehicle-treated contralateral limbs showed almost no response to the maximum force (300 g) used throughout the study, thus no significant PWT change was observed.

### Cytokine enzyme-linked immunosorbent assay (ELISA)

**Figure 2:**
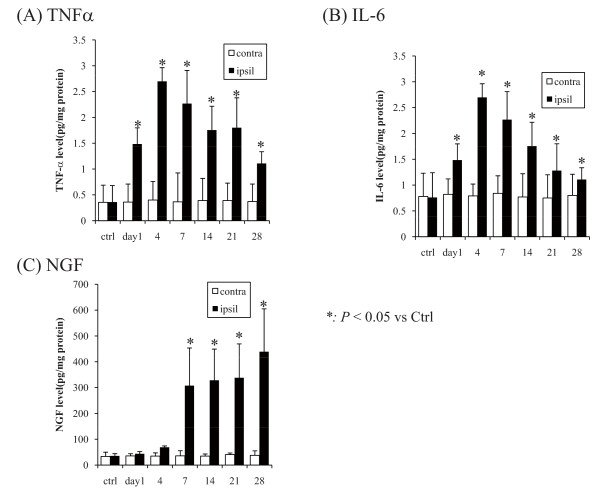
**Modified concentrations of cytokines**. (A) TNF-α, (B) IL-6, and (C) NGF. The concentrations of TNF-α and IL-6 were significantly elevated starting on day 1 after the injection and continued to increase with its peak on day 4, and they were gradually decreased as the postinjection period progressed (*P *< 0.05; Figure 2A, 2B). The concentration of NGF was significantly increased at 7 days after the injection (*P *< 0.05), and the increasing continued after this point (Figure 2C).

Figure 2 shows the modified concentrations of cytokines. The concentrations of TNF-α and IL-6 were significantly elevated starting on day 1 after the injection and continued to increase with its peak on day 4, and they were gradually decreased as the postinjection period progressed (Figure 2A, 2B). The concentration of NGF was significantly increased at 1 week after the injection (*P *< 0.05), and the increasing continued after this point (Figure 2C).

### Retrograde neurotracing and immunohistology of the DRG

**Figure 3:**
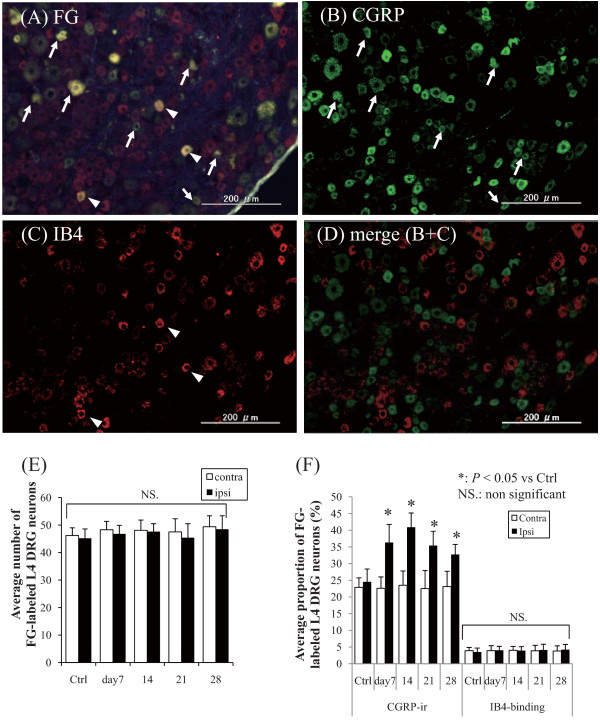
**Representative fluorescence photomicrographs of L4 DRG neurons**. The scale bars are 200 mm. (A) FG-labeled DRG neurons, (B) CGRP-ir DRG neurons, and (C) IB4-binding DRG neurons. All photomicrographs are from the same section. Arrows in A and B indicate FG-labeled CGRP-ir DRG neurons. Arrowheads in A and C indicate FG-labeled IB4-binding DRG neurons. FG-labeled DRG neurons were observed at all levels. (D) merged image acquired from 3(B) and 3(C). There were no overlaps between the CGRP-ir and IB4-binding DRG neurons (E) The average numbers of the FG-labeled L4 DRG neurons. The numbers did not significantly change between each time point. (F) Average proportion of FG-labeled CGRP-ir or IB4-binding DRG neurons. CGRP-ir or IB4-binding DRG neurons were observed at every level of DRG without significant deviation in each experimental group. The numbers of ipsilateral FG-labeled CGRP-ir DRG neurons were significantly increased after 7 days after the intraarticular injection, whereas the contralateral DRG showed no significant change in the expression of CGRP. The proportion of IB4-binding DRG neurons showed no significant differences among the groups.

**Figure 4:**
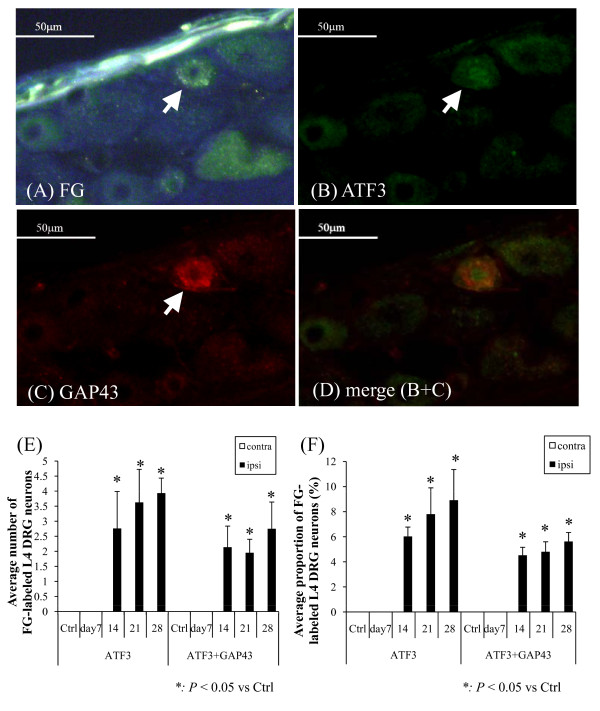
**Representative fluorescence photomicrographs of L4 DRG neurons**. (A) FG-labeled DRG neurons, (B) ATF3-ir DRG neurons, and (C) GAP43-ir neurons. (D) Distribution of FG-labeled ATF3-ir GAP43-ir DRG neurons. The scale bars are 50 μm. All photomicrographs are from the same section. The cytoplasm was immunopositive for FG and GAP43, whereas the nuclei were immunopositive for ATF3. The arrow indicates the same DRG neuron is positive for FG, ATF3-ir, and GAP43-ir. (D) Merged image acquired from Figures 4(B) and 4(C). In ATF3-ir GAP43-ir DRG neurons, the nuclei and cytoplasm are stained green and red, respectively (E) The average numbers of the FG-labeled ATF3-ir/ATF3-irGAP43-ir DRG neurons. The number of the appropriate cells was small but increased significantly after 14 days post-injection (F) The number of FG-labeled ATF3-ir DRG neurons was significantly increased in the ipsilateral MIA-treated knees (*P *< 0.05), and these neurons were significantly more prevalent 14 days after MIA injection (*P *< 0.05). FG-labeled ATF3-ir and GAP43-ir DRG neurons showed a similarly significant temporal increase (*P *< 0.05). There were no ATF3-ir or ATF3-ir GAP43-ir neurons in the contralateral side.

Figures 3 and 4 show typical fluorescence photomicrographs of processed L4 DRG neurons. FG-labeled DRG neurons, which innervate knee joints, were present at all bilateral DRG levels (Figure 3A, 3E). No lateral bias in the proportion of FG-labeled DRG neurons was observed at the same DRG level. Small-, intermediate-, and large-sized DRG neurons were included in each specimen and were counted along with the FG-labeled small-sized DRG neurons. Using a fluorescence microscope, both CGRP-ir DRG neurons and IB4-binding DRG neurons were observed (Figure 3B and 3C), and without any overlaps between these DRG neurons (Figure 3D), and the average numbers of the FG-labeled neurons did not show significant changes between each time point (Figure 3E). Figure 3F shows the average numbers of FG-labeled CGRP-ir or IB4-binding DRG neurons. The expression of CGRP-ir in ipsilateral DRG neurons had significantly increased after 7 days after the injection compared with the expression in the contralateral DRG neurons (*P *< 0.05), and the expression was greater at 14 days (*P *< 0.05). However, the proportion of IB4-binding DRG neurons showed no significant difference in their distribution throughout the postinjection period.

ATF3-ir DRG neurons were observed, but only in the ipsilateral DRG. No ATF3-ir DRG neurons were observed in the contralateral DRG throughout the experimental period. Significant numbers of ATF3-ir DRG neurons were observed in only the ipsilateral DRG after 14 days after the injection, and their proportions significantly increased as the postinjection period progressed (*P *< 0.05). FG-labeled ATF3-ir GAP43-ir DRG neurons showed a similar increase in their expression (*P *< 0.05).

### Immunohistology of the dorsal horn of the spinal cord

**Figure 5:**
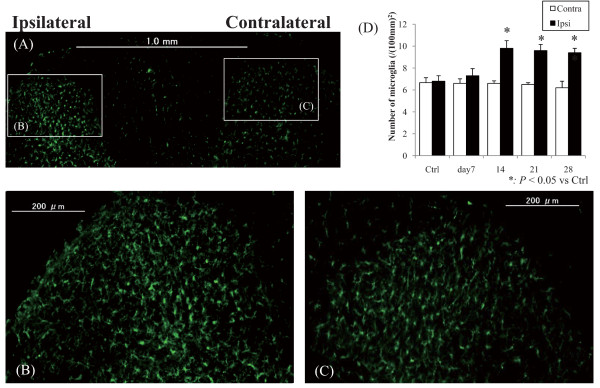
**Fluorescent photomicrographs of the dorsal horn of the spinal cord immunostained for Iba1 at 28 days after the injection **(A), (B) and (C) are magnifications of the 28 days specimen. Scale bars are (A) 1.0 mm and (B)-(C) 200 μm. Iba1-ir microglia were observed in bilateral dorsal horns (A), and those in the ipsilateral dorsal horn showed proliferation and hypertrophy relative to those on the contralateral side. (D) Quantified number of microglia. The ipsilateral dorsal horn microglia were significantly more abundant than those on the contralateral side at after 14 days after the injection (*P *< 0.05).

Figures 5 show typical fluorescent photomicrographs of the dorsal horn of the lumbar enlargement of the spinal cord immunostained for Iba-1 (Figure 5).

Iba1-ir microglia were observed in dorsal horns bilaterally (Figure 5A), and those in the ipsilateral dorsal horn exhibited proliferation and hypertrophy (Figure 5B) compared with those on the contralateral side (Figure 5C). The number of microglia in the ipsilateral dorsal horn was significantly greater than that on the contralateral side after 14 days after the injection, and it achieved a peak at day 14 and 21. (Figure 5D) (*P *< 0.05).

## Discussion

The present study showed that a rodent knee OA model induced by MIA exhibited tactile allodynia with decreased PWT and elevated proinflammatory mediators in local tissues. At the DRG level, the proportion of CGRP-ir DRG neurons innervating the knee joint significantly increased, unlike that of IB4-binding DRG neurons. Furthermore, the proportions of FG-labeled ATF3-ir and FG-labeled ATF3-ir GAP43-ir DRG neurons were significantly increased in the postinjection period. In the spinal cord, more proliferation of microglia were observed in the ipsilateral dorsal horn.

### Histological and behavioral evaluation of the MIA-induced OA model

MIA injection has been reported to cause joint pathology via the inhibition of glycolysis, thereby targeting avascular cartilage and causing chondrocyte death [30]. MIA-treated knees that were histologically evaluated in the present study exhibited osteoarthritic histological changes. The measurement of diameters of the knees increased significantly after day 7, which indicates the pathogenesis of knee OA in conjunction with the pathological findings The distribution of the weights of the animals showed no bias; therefore, the results of the von Frey tests indicate escape behavior and not lifting from physical loading. The significant decrease of PWT indicated tactile allodynia pathogenesis, consistent with a previous study [28]. This decrease is considered to result from allodynia derived from central sensitization; the increase in afferent signaling from the joint nociceptors to the spinal cord neurons results in increased sensitivity of the spinal cord neurons to input from the joint, rendering the spinal cord neurons hyperexcitable [31].

Some studies have noted "mirror pain" that could be observed as a referred pain in the contralateral limb [32], whereas the results of the present study did not reveal significant evidence for this. This implies significant laterality of the pathogenesis of OA pain-related activities in the ipsilateral knees compared with that in the contralateral knees.

### Cytokine expression in the local tissues of the knee joints

In the present study, the production of TNF-α and IL-6 showed significant temporal increases prior to the elevation of NGF production. MIA injection, by inducing chemical injury, might have induced the production, and inflammactivation of chondrocytes or synovial membranes, or both, is considered to play an important role as mentioned in the background. The evidence shows that normally quiescent chondrocytes, as well as synovial cells, respond to repetitive excess mechanical loading via stress-induced intracellular signals that mediate the production of proinflammatory mediators such as cytokines and cartilage-degrading proteinases [33]. Another study found that synovial inflammation is a factor that likely contributes to dysregulation of chondrocyte function, favoring an imbalance between the catabolic and anabolic activities of chondrocytes in remodeling the extracellular cartilage matrix [34]. Thus, the activated chondrocytes and synovial membranes should be the major sources of the elevated proinflammatory mediators, among which TNF-α produced from the activated chondrocytes or synovial membranes can induce IL-6 upregulation and neuropathic pain in the spinal cord and DRG [35]. In parallel, another study found that IL-6 enhances the expression of TNF-α receptors [36], thus these proinflammatory mediators act in concert to provoke further inflammation. NGF is generally reported to be involved in chronic inflammatory or neuropathic pain states [37]. NGF is physically produced in articular structures and expressed in normal and OA synovial tissues, being increased in synovial inflammation, especially upon synovial tissue exposure to TNF-α [14]. Thus, NGF production during inflammation might be involved in the modulation rather than in the induction of the joint inflammatory response.

The findings of the present study suggest that the elevated production of TNF-α and IL-6 in the acute phase after MIA injection may have induced elevated production of NGF and that these elevated proinflammatory cytokines are involved in the modulation of joint inflammation.

### Evaluation of sensory innervation at different DRG and spinal cord levels

The present study showed that sensory neurons innervating the knee are predominantly CGRP-ir. The average numbers of the FG-labeled neurons did not significantly change between each time point, which indicates that there was no significant increase or decrease of the neurons innervating the joint. CGRP-ir DRG neurons are reported to be NGF-dependent and critical to hyperalgesic responses induced by inflammation [38-40], which also means that these DRG neurons are "sensitive" to increased levels of NGF [16-18]. This may explain the evidence for increased numbers of CGRP-ir DRG neurons in the MIA-injected knees in the present study, which may result from stimulation of the peripheral nerve fibers by increased peripheral NGF production initiated from local inflammation as proposed previously.

The present study showed evidence for a low percentage of IB4-binding DRG neurons (less than 4%). This approximately coincides with the findings of a previous study that reported the sensory innervation of hip joints [29], while another study reported a complete absence of IB4-binding DRG neurons [41]. However, IB4-binding DRG neurons may not be involved in pain generation without any overlaps between CGRP-ir neurons.

The numbers of both ATF3-ir and ATF3-irGAP43-ir FG-labeled DRG neurons showed significant temporal increases compared with their levels on the contralateral side. ATF3 is not thought be expressed during inflammation [42], thus the increased expression of ATF3-ir in DRG neurons suggests the evidence of gradually progressive nerve injuries. The pain behavior evaluated in the hind paw using von Frey filaments is thought to be radiational pain derived from allodynia from the previous studies. Therefore, we should consider that it is not directly related to the neuronal damage indicating ATF3 or GAP43 immunoreactivity. However, the results of the present study suggest that both the behavioral changes and ATF3 production in DRG changes 7-14 days after the injection are related to each other.

Simultaneously, the significant gradual increase in the number of ATF3- and GAP43-ir DRG neurons implies a restoration process of the injured nerves in addition to the nerve injuries. The progressive nerve injury and regeneration may have resulted from the degradation of cartilage; the physical wearing of cartilage leads to exposure and degradation of subchondral bone, where sensory nerve ingrowth and pain-related mediators increase [43]. Subsequently, the exposed nerve endings may become physically injured, which can accelerate nerve ingrowth into the subchondral bone. This can lead to the temporally increased expression of ATF3 and GAP43. The absolute values of the proportions of ATF3-ir and ATF3-irGAP43-ir DRG are not very high. However, we consider its significant existence in the ipsilateral DRGs to be important. In stronger neuronal models such as the sciatic nerve axotomy model, ATF3-ir and GAP43-ir are frequently and clearly observed [44]. Therefore, we can hypothesize that MIA injection does not induce such drastic neuronal damage during the acute phase but the neuronal damage accompanying ATF3 immunoreactivity occurs as the neuronal damage proceeds.

Furthermore, it has been suggested that the proliferating microglia should also play a crucial role as is already mentioned in the background. Furthermore, increased levels of proinflammatory cytokines such as TNF-α and IL-6 produced after peripheral nerve injury have been reported to induce neuropathic pain by enhancing the activities of microglia in the spinal cord [45,46]. The present study showed significant proliferation of microglia in the ipsilateral dorsal horn in addition to the elevated levels of proinflammatory cytokines, which can contribute to the neuronal damage, which may lead to the pathogenesis of neuropathic pain. In addition, the changes in the dorsal horn of the spinal cord regarding microglia were significant only in the ipsilateral dorsal horn. In general, many changes in the nervous system can occur bilaterally even when there is a unilateral disease, which was not observed in the present study. One of the reasons for this is likely the post-injection period. Therefore, future investigations should be performed for longer periods

The findings above indicate that the MIA-induced OA pain results from inflammation, which initiates an inflammatory pain state, and subsequently, and neural damage gradually arising along with progressive cartilage degradation may induce a chronic state such as a neuropathic pain state. Also infrapatellar fat pad is reported to be a source of the pain in OA, thus we should take these reports into consideration [47]. Considering translational research, this may have important implications for targeted analgesic therapies. In addition to antiinflammatory therapies such as NSAIDs, which may be mainly effective for the early treatment of OA, treatment for neuropathic pain may be effective for OA patients in metaphase.

The present study has some limitations. First, MIA-induced OA is a chemically induced model. To clarify the details, further investigation with other nonchemically induced pathological models, such as partial medial meniscectomy, should be conducted. Second, the behavioral test for tactile allodynia with von Frey hairs can be somewhat inaccurate and depends on the responses of the animals. To analyze the behavior of rats objectively and correctly, it may be helpful to use an apparatus to analyze their behavior by using other methods such as evaluating weight bearing in free-moving walking animals [48] as well as response to heat or measuring direct pressure to knees [49]. Third, the samples from each knee were complex structures consisting of synovium and capsules, and we did not investigate the distribution of proinflammatory cytokines in each structure. The distribution of receptors and their expression as well as cytokines themselves should be examined in future studies. Fourth, the present study examined up to 4 weeks after MIA injection. As the neuropathic pain state has a tendency to increase temporally, the behavior of the inflammatory pain state may be important; therefore, further investigation with a longer postinjection period may be needed. Lastly, we examined the knee joints to confirm the visual absence of yellow leakage of FG from and around the knee joints throughout the experiments. However, we did not examine this nor circulation using fluorescence microscopy. We think the effect of the leakage is counteracted by averaging the data. Further study may be necessary to clarify whether any leakage actually occurred.

## Conclusions

The present study showed the characteristics and the time course of pain-related sensory innervation in a MIA-induced rodent knee OA model during a 28 days postinjection period. The OA knee joint was predominantly innervated by CGRP-ir DRG neurons, which were elevated in number in the postinjection period, peaking after 14 days along with significant decreases in PWT. Elevated numbers of proinflammatory cytokines in the local tissue suggested the initiation of local inflammation. At the same time, the gradual elevation in the number of ATF3-irGAP43-ir DRG neurons and the gradual proliferation of microglia in the dorsal horn of the spinal cord demonstrated progressive nerve injury, which can suggest the gradual initiation of the neuropathic pain state along with the inflammatory pain state.

## Competing interests

The authors declare that they have no competing interests.

## Authors' contributions

SO, TI, and MM designed and performed all of the experiments, analyzed data, and drafted the paper. NO, GI, YE, HK, GA, TT, YA, TK, KT, and SO supervised the project and edited the manuscript. All authors contributed to data interpretation and have read and approved the final manuscript.

## Pre-publication history

The pre-publication history for this paper can be accessed here:

http://www.biomedcentral.com/1471-2474/12/134/prepub
